# Physiological and Molecular Mechanisms of Saline–Alkali Stress Affecting Rice Growth and Regulation of Stress-Resistant Cultivation

**DOI:** 10.3390/plants15213474

**Published:** 2026-11-06

**Authors:** Guanglong Zhu, Chenghao Hu, Qingqing Geng, Qiwei Yang, Sizhuo Chen, Yuewen Ding, Hussam Ahmad, Tiankang Zhu, Wen Sun, Irshad Ahmad, Guisheng Zhou

**Affiliations:** 1 Joint International Research Laboratory of Agriculture and Agri-Product Safety, Ministry of Education of China, Yangzhou University, Yangzhou 225009, China; zhuguang2007@163.com (G.Z.);; 2 Zhongshan Biological Breeding Laboratory, Agricultural College, Yangzhou University, Yangzhou 225009, China; 3 Jiangsu Co-Innovation Center for Modern Production Technology of Grain Crops, Agricultural College, Yangzhou University, Yangzhou 225009, China; 4 College for Overseas Education, Yangzhou University, Yangzhou 225009, China

**Keywords:** saline–alkaline stress, rice yield formation, grain quality, regulation

## Abstract

Arable land is diminishing rapidly as a result of environmental degradation and urbanization. The development and utilization of saline–alkali soils therefore represent an important strategy for ensuring global food security. Rice (*Oryza sativa* L.) is a staple food for over half of the global population, yet its productivity is severely constrained by saline–alkali stress. Salinity (mainly NaCl) and alkalinity (high pH caused by Na_2_CO_3_/NaHCO_3_) markedly impair both yield formation and grain quality. This review establishes diagnostic thresholds for salinity (ECe) and alkalinity (pH) to mitigate their adverse effects. The effects of saline–alkali stress on rice germination, seedling establishment, plant growth, reproductive development, and grain filling are summarized. And the underlying physiological and molecular mechanisms are elucidated, including ion homeostasis (mediated by *OsHKT1;5*, *OsSOS1*, and *OsNHX1*), osmotic adjustment, as well as alkalinity-specific processes such as rhizosphere acidification, organic acid exudation, and Fe/Zn acquisition under high pH, endogenous hormone crosstalk, antioxidant defense, starch/protein biosynthesis, and related key genes and quantitative trait loci (QTLs). Furthermore, recent advances of stress-resistant cultivation and regulation strategies are highlighted. On this basis, future research directions are prospected. This review provides a comprehensive understanding of the physiological and molecular mechanisms underlying rice saline–alkali tolerance, with important implications for achieving high yield and quality in rice production on saline–alkali soil.

## 1. Introduction

Rice (*Oryza sativa* L.) is one of the most important staple food crops of the world, consumed by more than 50% of the world population. But environmental degradation and urbanization are limiting the production of rice [1]. Globally, more than 800 million hectares of land are affected by salinity [2], and 20–30% of irrigated land suffers from salt stress [3,4]. Only in Asia, salinization causes 20–30% rice yield losses, and these losses are intensifying with ongoing sea-level rise [5]. The saline–alkaline stresses are widespread in arid and semi-arid regions and coastal areas, and have become a major environmental constraint on rice production [6]. A central message of this review is that salinity, alkalinity, and sodicity are physiologically and agronomically distinct stresses and should not be pooled into a single “salt stress” category. Each stress imposes different primary constraints, activates different adaptive mechanisms, and requires different management and breeding approaches. The commonly used umbrella term “saline–alkali stress” should therefore be used with caution.

Rice shows considerable intraspecific modification in tolerance to salinity (excess neutral salts, especially NaCl) and alkalinity (excess Na_2_CO_3_/NaHCO_3_ and high pH) stresses, although it is generally considered a salt-sensitive crop [7]. Under salinity stress, growth and yield reduction can be detected at approximately 30–40 mM NaCl (ECe ≈ 3–4 dS m^−1^) in sensitive genotypes [8,9], and yield losses exceeding 50% have been reported where ECe > 10 dS m^−1^ [10]. These values should be regarded as indicative rather than as universal boundaries, because the effective threshold depends on genotype, growth stage, soil texture, and water management (Table 1). Reductions in tillering, spikelet fertility and grain filling are the main contributors [7] to the yield reduction. Alkalinity stress (pH > 9) primarily causes problems with nutrient use (particularly Zn, Fe and P) and soil structure [7,11]. Salinity is caused by excess soluble salts, primarily NaCl and Na_2_SO_4_, that increase the electrical conductivity of the soil solution (ECe) without necessarily raising pH. Alkalinity is caused by high soil pH (typically > 8.8) due to the presence of Na_2_CO_3_ and NaHCO_3_, which cause the immobilization of Fe, Zn and P and aggregate dispersion [12]. Sodicity is the result of the soil cation exchange complex being dominated by Na^+^, represented by the ESP (exchangeable sodium percent-age), which is alkaline in most cases but can be neutral [13] Although salinity and alkalinity often co-occur, they impose distinct primary constraints [7]. Salinity is dominated by osmotic stress and Na^+^/Cl^−^ toxicity, whereas alkalinity is driven by high pH, bicarbonate/carbonate toxicity, Ca^2+^ deficiency, and Fe/Zn/P immobilization. Sodicity, in turn, leads to main degradation of soil structure and poor water infiltration. Therefore, salinity-specific, alkalinity-specific, sodicity-specific and shared adaptive mechanisms should be distinguished when interpreting rice tolerance and designing management or breeding strategies [13]. In strongly alkaline soils (pH 9.5–10), rice roots are largely damaged, causing root severe stunting, then water uptake is inhibited [14,15], and ultimately grain yield declines from 25% to 65% [16,17].

In addition, saline–alkaline stress also influences grain quality [18,19]. Salinity effects photosynthesis transport to the panicle and alters starch and protein deposition in the endosperml [20,21]. This has led to the reduction in grain size coupled with increased chalkiness and higher protein/starch ratio [22,23]. The rice grains are usually chalky, discolored, brittle grains, alongside poor cooking and eating qualities [1,24]. In addition, the rice grains have higher protein but lower amylose and poorer milling quality under salt stress [2,25]. For instance, the head rice yield and amylose of the rice grain decreased while the chalkiness of the rice grain increased with salinity conditions in reproductive stage from 5–12 dS/m [2,26]. However, the effect of salinity on grain quality is not same: it relies on stress intensity, environmental situation, and genotype, and mild stress may even improve some quality traits.

Therefore, improving rice yield and quality is important on saline–alkaline land. This review explores the adverse effects of saline–alkaline stress on yield formation, yield quality from agronomic, physiological and genetic aspects. We review the diagnostic criteria (Table 1) and describe the regulatory networks underlying the responses to stress during development, during ion transport, in hormone signaling, and in carbon and nitrogen allocation. A list of key genetic loci (Wx; SSIIa) and QTLs are compiled (Table 2). At the same time, agronomic management practices and breeding strategies are adopted to reduce saline–alkaline stress. This study provides important implications for the sustainable development of high-yield and high-quality rice production in saline–alkaline soils.

**Table 1:** Indicative diagnostic thresholds for salinity, alkalinity and sodicity in rice cultivation. Values are compiled from the cited sources and are indicative, not universally applicable; actual thresholds vary with genotype, growth stage, soil texture, climate and management.

Parameter	Category	Threshold	Implication for Rice Yield (Approximate)	
Soil salinity (ECe)	Non-saline	ECe < 4 dS/m	No significant salinity stress	[27,28]
	Slight	ECe 4–6 dS/m	Slight yield reduction, mainly in sensitive varieties	[8,29]
	Moderate	ECe 6–10 dS/m	Moderate yield reduction	[10]
	High	ECe > 10 dS/m	Severe yield reduction; most conventional varieties fail	[10]
	Good quality	EC< 1.0 dS/m	Little risk of salinity buildup (ideal for rice)	[30]
Irrigation water salinity (EC)	Moderate	EC 1.0–2.5 dS/m	Manageable with leaching; sensitive varieties may show slight yield decline (1 t/ha yield loss under floodwater EC > 2 dS/m)	[30]
	High	EC > 2.5 dS/m	High risk requires salt-tolerant varieties and soil leaching; high yield reductions	[30]
Soil alkalinity (pH)	Non-alkaline	pH < 8.8	No direct alkalinity stress (flooded paddy soil pH 7)	[6]
	Slight	pH 8.8–9.4	Mild nutrient deficiency begins, slight yield impact	[31]
	Moderate	pH 9.5–9.7	Serious nutrient deficiency, soil dispersion, moderate yield reduction	[16,17,32]
	High	pH ≥ 9.8	Severe alkalinity; most rice varieties fail to grow (only tolerant genotypes yield)	[16,17,32]
Sodicity	ESP%	< 15	Non-sodic	[27,28]
		15–30	Sodic; soil dispersion and poor water infiltration	[28]
		> 30	Strongly sodic; severe structural degradation	[28]
	SAR	< 13	Non-sodic (approximate)	[27,28]

Note: ECe, electrical conductivity of soil saturation extract. ECw, electrical conductivity of irrigation water; ESP, exchangeable sodium percentage; SAR, sodium adsorption ratio. The salinity–yield classes follow the widely used ECe 3–4 dS m^−1^ threshold for rice [8,10]; pH classes follow IRRI guidance for alkali stress [7]. In flooded paddies, soil pH tends to drift toward neutrality, which partly mitigates alkali stress [25]. All values are indicative and should be calibrated locally.

## 2. Effects of Salt–Alkali Stress on Rice at Different Growth Stages

The saline–alkaline stress occurs at all the growth stages of rice, and the tolerance to saline–alkaline stress is various at different growth stages [33,34]. Generally, rice is most sensitive to saline–alkaline stress at the seedling and reproductive stage and is relatively tolerant at mid-vegetative growth stage [29].

### 2.1. Germination and Early Seedling Stage

In salinity–alkaline soils, high salinity and nutrient deficiency are major constraints for seed germination. Salinity inhibits water imbibition and promotes cytotoxic ion accumulation, leading to abnormal seedling establishment or mortality [35,36]. Both germination rate and the elongation of the radicle may significantly decrease under moderate salt concentrations (40 mM NaCl, EC 4 dS/m). Under highly saline conditions (ECe > 8 dS m^−1^), no germination is observed in salt-sensitive rice varieties, while salt-tolerant genotypes show only partial germination [37]. These adverse effects are further exacerbated under alkaline conditions, which are especially detrimental to seed metabolism and energy supply for embryonic growth [38]. In addition, chlorosis frequently occurs in newly emerged coleoptiles, primarily due to insufficient available iron under saline–alkali stress [39].

During the first 2–3 weeks after germination, rice seedlings are more sensitive to salt stress. Zeng and Shannon [9] reported that rice shoot growth was significantly reduced at a salinity level of 2 dS m^−1^, with a 50% decrease in growth rate at 6 dS m^−1^. Likewise, alkaline stress (pH 9–10) leads to plant stunting, root browning, leaf tip necrosis and chlorosis of seedling, and limited leaf expansion [12]. Alkaline-stressed seedlings also suffer from micronutrient deficiency [39]. Consequently, poor seedling establishment is prevalent in salt-affected soils [30]. Thus, high-quality seedling populations are the basis for high yield on saline–alkali land. These germination-stage effects are stress-type specific: salinity mainly delays imbibition through osmotic and ionic constraints; alkalinity additionally impairs seed metabolism through high pH and micronutrient deficiency; sodicity acts indirectly through soil crusting and poor water infiltration.

### 2.2. Vegetative Stage (Tillering to Panicle Initiation)

After transplanting, rice plants may gradually acquire saline tolerance, and some genotypes can withstand moderate salt levels (ECe 4–6 dS/m). In this time period, rice produces more osmotic adjustment substances (e.g., proline, sugars) and sequesters Na^+^ in older leaves or vacuoles, reducing salinity stress [40,41]. Nevertheless, plant height, tiller number, biomass and grain yield are still significantly reduced under salinity stress during vegetative growth [1,42]. It was demonstrated that at saline irrigation (ECe 6–8 dS/m) during the vegetative stage the number of tillers and biomass reduced by 30% and 50%, respectively [10]. Under sodic soils with high pH values, root growth is limited and shallow [13]. Furthermore, nutrient uptake (particularly phosphorus and micronutrients) is limited under alkaline conditions [43]. Typical phenotypic traits during the vegetative stage under saline conditions include dark green with thickened leaves, midday leaf rolling, and browning and dieback of older leaves from the tips [43,44]. Rice was also observed to have 50% reduction in plant height and tiller numbers with the increase in soil pH to 9.5 at 5 dS/m ECe [32]. Overall, saline–alkali stress during the vegetative growth stage primarily reduces tiller number, thereby decreasing panicle number and causing yield reduction.

### 2.3. Reproductive Stage (Booting, Flowering, Anthesis)

Rice is generally most sensitive to salt stress during reproductive stage, particularly from the panicle initiation to the booting period, although the level of sensitivity differs among genotypes [18,19]. At the booting stage, panicle differentiation is inhibited and induces spikelet sterility under salinity stress [45]. Salt stress during the young microspore stage causes the reduction in pollen formation and defective anther dehiscence which results in a high percentage of spikelet sterility [20]. Physiologically, salt stress limits the supply of carbohydrates to developing panicles. It also decreases gibberellin levels, leading to hormonal imbalance. These effects ultimately cause abnormal development of floral organs [21]. At the flowering stage, excessive salt stress may cause dehydration of stigmatic tissues and reduce the growth of pollen tubes, which may also inhibit the successful pollination process [22]. However, under alkaline stress, high soil pH environments aggravate micronutrient deficiencies at the reproductive stage [23]. For instance, zinc deficiency commonly occurs in alkaline fields, which is the main reason for spikelet sterility and malformed panicles [24,25]. Previous studies have noted that a salinity of > 4–6 dS/m at flowering will reduce grain yield by 50–80% [18,26]. Even in tolerant varieties, the grain yield sharply declines under continued salinity during flowering [46]. The number of filled grains per panicle was reduced by 70% under salt stress (ECe 5 dS/m) during the panicle initiation stage, but there was only a 30% reduction under the same stress from tillering to panicle initiation [33]. This result confirms that rice exhibits greater susceptibility during the reproductive stage.

### 2.4. Grain Filling and Maturation

**Figure 1:**
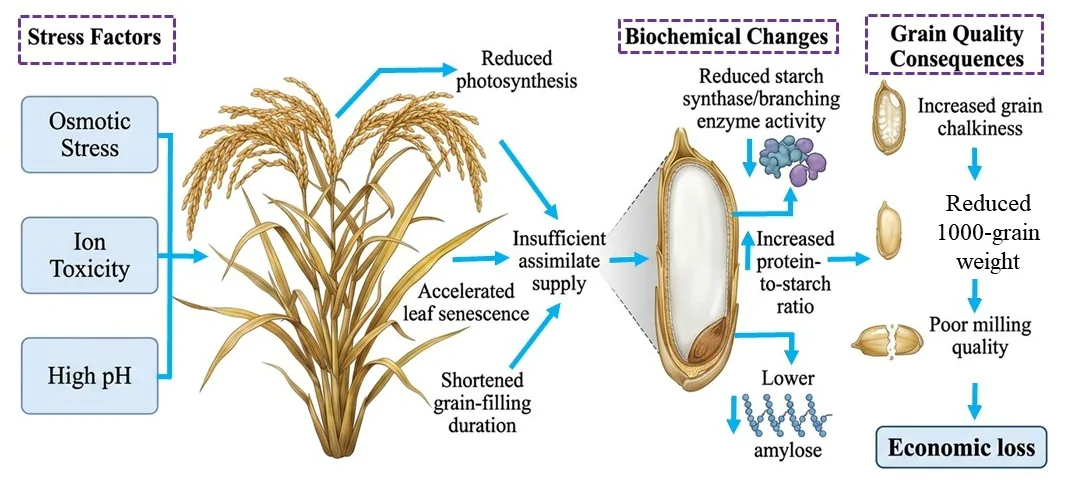
The effects of saline–alkalinity on grain quality of rice. Stress factors such as osmotic stress, ion toxicity, and high pH trigger biochemical changes, including reduced photosynthesis, insufficient assimilate supply, accelerated leaf senescence, and shortened grain-filling duration. These changes lead to specific grain quality consequences such as increased chalkiness, reduced kernel size and weight, poor milling properties, lower amylose content, an elevated protein-to-starch ratio, and decreased starch synthase activity. Ultimately, these quality impairments result in economic loss.

The effect of salinity on grain quality is therefore not the same; it is a response that relies on stress intensity, environmental conditions, and genotype [47]. The grain-filling and physiological maturity stage remain sensitive to saline–alkaline stress, which mainly diminishes spikelet size, grain weight and quality (Figure 1). The grain-filling period is shortened during this period, due to increased senescence of leaves and reduced photosynthesis [18,19]. Under saline–alkaline stress, deficit assimilation results in delayed grain development and reduces filling rate causing unfilled grains and poor appearance quality [18,19]. Furthermore, salt stress tends to promote protein/water accumulation and chalkiness of grain, which results from the down-regulation of starch biosynthesis genes, starch synthase and starch branching enzyme, which are more sensitive to high salinity than the genes responsible for protein accumulation [48,49]. A previous study confirmed that rice subjected to 17–34 mM NaCl (1.5–3 dS/m) increased chalkiness and reduced 1000-grain weight [1]. However, alkaline stress (high pH) exacerbates premature senescence and incomplete grain filling by nutrient deficiencies (Zn, N).

Interestingly, mild salt stress (ECe 2–3 dS/m) can sometimes enhance quality traits, such as improving grain translucency, reduced amylose content or increasing head rice yield [1]. Recent studies revealed that low concentration (0.1%) of salt stress resulted in enhancement in milling and eating quality in three japonica cultivars; however, it reduced grain yield by 20% [2]. The possible reason was that endosperm composition was altered, which resulted in reduced amylose and higher gelatinization temperatures [50]. Under moderate and severe stress (≥0.2% soil salt or 5 dS/m), grain quality significantly decreases, like chalkiness and brittleness increased, and palatability decreased [2]. Saline–alkaline stress during grain filling ultimately reduces yield and market value, primarily through increased chalkiness, altered cooking profiles and elevated protein content [2,51] (Figure 1). Mild stress (about 2–3 dS/m) may enhance attributes in genotypes, but usually at the cost of yield, and this benefit is lost under moderate to severe stress (≥5 dS/m) [52].

## 3. Mechanisms of Rice Response to Saline–Alkaline Stress

**Figure 2:**
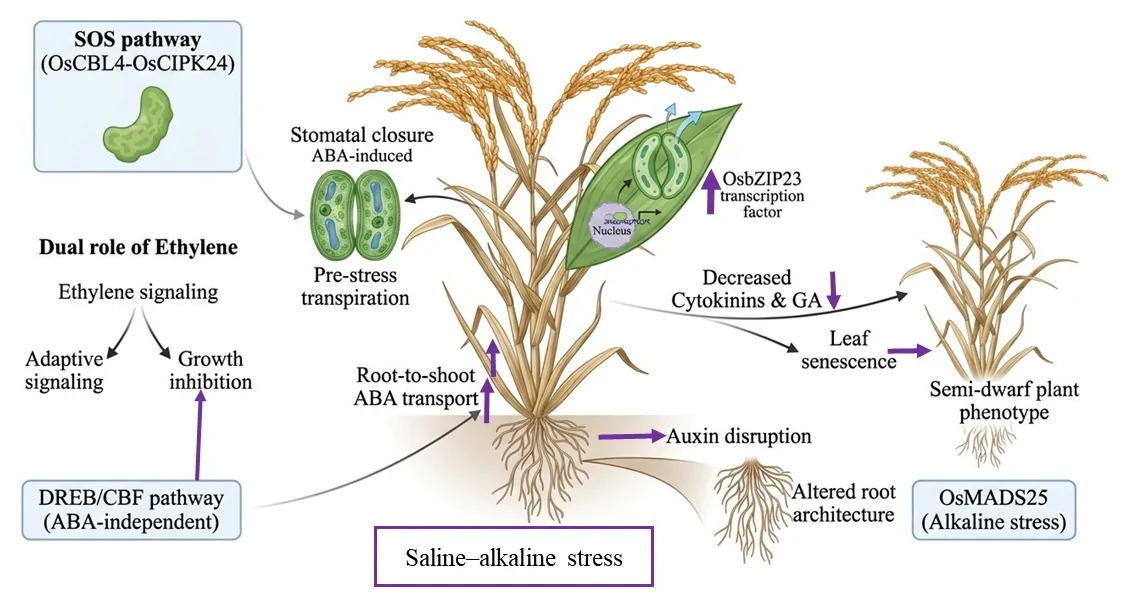
Overview of signaling and adaptive responses of rice under saline–alkaline stress. Key components include the SOS pathway (OsCBL4-OsCIPK24), the dual role of ethylene in stress signaling, upregulation of the OsbZIP23 transcription factor, reduced cytokinin levels and gibberellins (GA), leaf senescence, auxin disruption leading to altered root architecture, OsMADS25 (induced by alkaline stress), root-to-shoot ABA transport, pre-stress transpiration changes, growth inhibition, and the ABA-independent DREB/CBF pathway. These interconnected pathways collectively mediate plant adaptation to saline–alkaline conditions.

Rice responses to salinity, alkalinity and sodicity involve both shared and stress-specific metabolic pathways. Under saline–alkaline environment, rice plants induce several physiological and molecular metabolic pathways to cope with the stresses. These key pathways include ion homeostasis and compartmentalization, osmotic adjustment, hormonal signaling networks, antioxidant defense systems, and crucial metabolic reprogramming (particularly concerning starch and protein deposition in grains), which are summarized in this section (Figure 2). Furthermore, the key genes and signaling networks were analyzed in Table 2.

### 3.1. Ion Homeostasis and Salt Exclusion

Rice is salt-sensitive due to its ability to absorb high amounts of Na^+^ and its poor ability to exclude Na^+^ from the roots [43]. Maintenance of ion homeostasis is important for preventing excessive Na^+^ and Cl^−^ accumulation and preserving K^+^ and nutrient uptake in rice under saline conditions. Tolerant rice genotypes can effectively restrict Na^+^ influx and root-to-shoot transport, or sequester Na^+^ into vacuoles, which can protect photosynthetic tissues; however, this capacity varies widely among verities and is influenced by environmental conditions [53,54]. This method is regulated by the *OsHKT1;5* (known as *SKC1*) gene (Table 2). This gene is a major QTL for salt tolerance: elite alleles found in tolerant landraces (Pokkali) can maintain a high K^+^/Na^+^ ratio in leaves to protect cellular function [55,56]. *OsHKT1;5* encodes a Na^+^ transporter that retrieves Na^+^ from the xylem sap back into root stelar cells, which can effectively limit shoot Na^+^ loading and reduce foliar accumulation [55]. Rice activates the SOS pathway (Salt Overly Sensitive) that controls plasma membrane Na^+^/H^+^ antiporter *OsSOS1*, which removes Na^+^ from root cells to the rhizosphere by using the proton gradient [57]. Overall, the interaction between *OsHKT1;5* (vascular unloading) and *OsSOS1* (cellular efflux) is likely to reduce net Na^+^ translocation to the rice plant shoots. It should be emphasized, however, that most evidence for the HKT/SOS-mediated Na^+^ exclusion model has been obtained under NaCl-dominated salinity. Whether the same mechanisms are equally effective under alkaline or sodic conditions remains uncertain. For example, several mapping populations that show strong Saltol effects under saline conditions fail to detect the same QTL when screened in alkali-stressed fields, and overexpression of *OsSOS1* or *OsNHX1* has repeatedly caused growth penalties under non-stressed conditions. Thus, although ion-homeostasis genes are well-established components of salinity tolerance, their value as universal breeding targets for all three stress types—salinity, alkalinity, and sodicity—requires further validation in stress-specific genetic backgrounds. In contrast, sensitive genotypes are unable to effectively lower Na^+^ resulting in toxic levels of Na^+^ buildup in leaves [58,59]. For the tolerant cultivar, Nonabokra, it was shown that it overexpresses *OsHKT1;5* genes, which assist in maintaining Na^+^ levels in the leaves at about 50% lower than in the susceptible cultivars [60].

Meanwhile, maintaining K^+^ homeostasis is very important under salt stress. Salinity causes K^+^ efflux via depolarization-activated channels [61,62,63]. In a salinity environment, high-affinity transporters (*OsHAKs*) and channels (*OsAKT1*) are activated in tolerant varieties to sustain K^+^ acquisition [64]. In addition, the SOS signaling module (particularly the *OsCBL4-OsCIPK24* (*OsSOS3-OsSOS2*) complex) regulates Na^+^ and K^+^ homeostasis by activating *OsSOS1*, which can facilitate Na^+^ efflux and prevent K^+^ leakage [65,66]. Alkaline stress also leads to Ca^2+^ deficiency and membrane destabilization, which can be prevented by the exogenous application of Ca^2+^ or gypsum [67]. Furthermore, tolerant genotypes exhibit constitutive upregulation of H^+^-ATPases, which supplies the electrochemical gradient required to energize antiport activity [68]. The ion-homeostatic mechanisms described above are largely NaCl-specific. Under alkaline stress, the primary constraints are high-pH-driven Fe/Zn deficiency and impaired rhizosphere acidification rather than Na^+^ toxicity per se. Under sodic stress, poor soil structure and waterlogging further complicate these responses. Thus, breeding solely for Na^+^ exclusion may be insufficient for alkaline and sodic fields, and stress-type-specific breeding strategies are required.

### 3.2. Osmotic Stress and Compatible Solute Accumulation

Higher salinity stress reduces the water potential and rice immediately experiences osmotic constraints that reduce root water uptake [43,69]. To alleviate osmotic stress effects, rice accumulates compatible solutes (including proline, soluble sugars, amino acids, etc.) to counteract external osmotic pressure [45,70]. Osmotic adjustment is the process of accumulation of solutes that help decrease the osmotic potential of cells and consequently aid in maintaining turgidity pressure and water uptake under stress conditions [71]. In contrast, osmoprotection refers to the stabilization of cellular structures and proteins against stress-induced damage, while compatible solutes can contribute to both processes. Among them, proline acts as an osmolyte and a molecular chaperone that stabilizes macromolecules [72]. Proline accumulation improves cellular homeostasis and salt resilience [73]. However, increased proline level demonstrates effective osmotic adjustment as well as changes in osmotic potential, plant water status, and turgor maintenance. There is a significant difference between cultivated rice and wild rice in the production of osmolytes as the wild rice accumulates glycine betaine, whereas the cultivated varieties accumulate alternative osmolytes, owing to a mutation in the BADH2 gene [74,75]. A persistent controversy in the literature is whether proline accumulation is an adaptive tolerance mechanism or merely a stress-induced symptom. In some sensitive rice genotypes, proline levels exceed those of tolerant genotypes under severe stress, suggesting that bulk proline content alone cannot serve as a reliable breeding marker. Similarly, the BADH2-dependent glycine betaine pathway is frequently described as osmoprotective, yet most cultivated rice varieties carry a non-functional badh2 allele, and the evidence that natural BADH2 variation translates into consistent yield or survival advantages under field salinity is weak. Most supporting data come from transgenic lines expressing heterologous BADH genes or from controlled-environment experiments. We therefore treat glycine betaine as a contributing osmoprotectant rather than as a determinant of field-level salinity tolerance. Generally, salt-tolerant cultivars accumulate higher concentrations of osmotic adjustment substances as compared to sensitive genotypes, thereby maintaining higher leaf hydration and turgor [41]. In terms of morphological characteristics, stomatal closure is activated to conserve water under saline and alkaline stresses [36]. Stomatal closure is not an isolated response but part of an integrated sequence involving soil/solution, root, hydraulic conductance, leaf water status, ABA and/or hydraulic signaling, stomatal conductance, and CO_2_ assimilation [76]. A lower soil solution water potential reduces root water uptake and hydraulic conductance, which lowers leaf water status. Hydraulic signals and/or ABA then reduce stomatal conductance, which in turn limits CO_2_ assimilation. However, this defense method prevents CO_2_ influx and inhibiting photosynthesis [40,77]. Furthermore, deep rooting conferred by the DRO1 locus offers rice to exploit deeper, less saline soil layers [78]. Organic acids secreted by roots can improve osmotic regulation and effectively alleviate saline–alkali stress [79]. Moreover, modern biotechnology enhances crop’s osmotic adjustment capacity and stress tolerance via genetic improvement and cultivation techniques [73,80].

### 3.3. Hormonal and Signaling Responses

Endogenous hormone balance and signaling pathways of rice are rapidly disturbed under saline and alkaline stresses. Abscisic acid (ABA) is one of the main hormones of stress response. ABA is produced in roots under salt stress and moved to shoots where it plays a role in stomatal closure and induction of stress-responsive genes [18]. Salt-tolerant rice cultivars often rapidly accumulate more ABA in order to conserve water and activate early genes defense [19]. Some genes in the ABA biosynthesis and signaling pathway (such as NCED enzymes or *PP2C/SnRK2* components) have been demonstrated to be involved in rice salt stress signaling [18]. For example, OsbZIP23 is a transcription factor that upon overexpression results in the upregulation of genes that are required for salt tolerance [21].

Moreover, ethylene production is enhanced under stress. The ethylene hormone plays a dual role in stress responses: appropriate concentration of ethylene can synergize with ABA to fine-tune stomatal aperture and activate adaptive signaling. The higher accumulation of ethylene in plants inhibits cell expansion and growth, triggering leaf epinasty and premature senescence [81,82]. Salt-tolerant genotypes produce less ethylene under stress compared to salt-sensitive genotypes. Plant growth-promoting rhizobacteria that produce ACC deaminase can reduce ethylene levels to alleviate salinity stress in wheat and rice [18]. The dual role of ethylene is highly context-dependent. Ethylene can induce adaptive responses, including stomatal closure, antioxidant defense and root elongation, at low to moderate concentrations and when it is first encountered during stress. However, when ethylene accumulates excessively or persists for prolonged periods, it inhibits cell expansion, accelerates leaf senescence, and reduces grain filling. In rice, rhizobacteria that can degrade ACC may be more effective at lowering ethylene production under salt stress, while salt-tolerant genotypes can also produce lower ethylene under salt stress [83,84,85]. Thus, the timing, concentration, and tissue specificity determine whether ethylene acts positively or negatively during stress adaptation. These contrasting observations illustrate that bulk hormone measurements at a single time point have limited predictive power. ABA also shows a dual, concentration-dependent role: rapid accumulation conserves water and activates early defense genes, but prolonged elevation accelerates leaf senescence and can reduce yield. Future work should therefore focus on tissue-specific, time-resolved hormone dynamics and on the spatiotemporal overlap of ABA, ethylene, and cytokinin signaling under each of the three stress types.

Moreover, under salinity, the production of cytokinin (CTK) is affected generally in a negative way, leading to further growth suppression and senescence of older leaves. Previous studies demonstrate that exogenous cytokinin treatment can delay leaf senescence and extend photosynthesis duration in salt-stressed rice [18]. Likewise, gibberellins (GAs) are also considerably decreased under salt-stress conditions. When the GA production is reduced, plants are shorter, internode length is limited, and yields are reduced [19].

In addition, low salinity levels negatively affect the distribution of Auxin (IAA) and cause the loss of root gravitropism and architecture under cadmium stress, although direct investigation under saline–alkaline stress in rice remains less documented [86]. The *OsGH3-2* gene under cadmium stress has been demonstrated to maintain auxin homeostasis to support continued root exploration; however, its relevance to salt stress needs further validation [86]. Other signaling molecules, such as brassinosteroids (BRs) and salicylic Acid (SA), provide tolerance through boosting the antioxidant defense and ionic stability [87].

In addition, melatonin (N-acetyl-5-methoxytryptamine) has emerged as an important regulator of plant responses to saline–alkaline stress [51]. Melatonin is synthesized from tryptophan via the same pathway that supplies auxin and serotonin, and although its classification as a phytohormone is still in an early stage, melatonin is believed to be a master regulator that integrates several stress-responsive pathways [88]. In rice, exogenous melatonin application enhances germination and seedling establishment under salt stress, delays salt-induced leaf senescence, and increases photosynthetic rate, biomass and grain yield under saline conditions [89]. These effects are related to (i) maintenance of ion homeostasis (reduced shoot Na^+^ accumulation, higher K^+^/Na^+^ ratio [90]); (ii) enhancement of antioxidant defense (increase in SOD, CAT and APX activities, decrease in H_2_O_2_ and MDA contents [91]); (iii) increase in accumulation of compatible solutes (proline and soluble sugars); and (iv) modulation of endogenous hormone balance (ABA and GA) [92]. Melatonin also acts as a direct ROS scavenger and interacts with ABA, auxin, GA, BR and ethylene signaling, indicating that it functions within, rather than separately from, the hormone network [93]. In addition, melatonin is linked to alkaline stress tolerance and alleviates Fe-deficiency-induced chlorosis, indicating special significance under sodic conditions [93]. Practical applications include seed priming and foliar spraying, which are simple and low-cost; however, optimal dose, application timing and genotype-specific responses still require further study.

All these hormone signals ultimately end up in a complex network that controls the expression of stress-related genes. Several key transcription factor families mediate the stress responses in rice. For example, DREB (CBF) proteins are induced by salt via ABA-independent pathways and activate osmotic stress-tolerance genes [18]. Overexpression of rice NAC and bZIP transcription factors, such as SNAC1 and OsbZIP72, enhances salinity tolerance, primarily by regulating downstream drought/salt-responsive genes and ROS-scavenging enzymes [19]. Rice CBL-CIPK networks (with a quantity of CBLs and CIPKs) respond to different stresses, and *OsCIPK24/SOS2* is specific to salt stress and interacts with OsCBL4 [20]. A MADS-box transcription factor, *OsMADS25*, has been reported to be upregulated under alkaline stress and has been proposed to contribute to root-system maintenance under high pH [21]. Its expression also differs between saline and alkaline treatments [7]. However, the functional evidence for *OsMADS25* is still limited: the principal supporting studies focus on root architecture, and the mechanistic link to alkalinity per se—as distinct from general salt, ABA or ROS-related responses—has not been established by loss-of-function or allelic analysis. We therefore describe *OsMADS25* as a alkalinity-responsive locus candidate rather than an alkalinity-specific gene. In addition protein kinases such as SnRK1 in rice are energy sensors and can help to modulate metabolism during salt stress [22].

### 3.4. Oxidative Stress and Antioxidant Defense

Under saline and sodic stresses, reactive oxygen species (ROS) are better accumulated in rice, including superoxide anion (O_2_^∙−^), hydrogen peroxide (H_2_O_2_) and hydroxyl radicals (∙OH) [94]. These ROS are mainly generated by dysfunctional photosynthetic electron transport (due to CO_2_ limitation), by dysfunction of mitochondria and by activation of NADPH oxidases [95]. Excessive ROS cause a great deal of harm to the cells through lipid peroxidation, oxidation of proteins, and degradation of nucleic acids [96].

Rice crop has two types of scavenging systems, including an enzymatic scavenging system and non-enzymatic scavenging system. Antioxidant enzymes include superoxide dismutase (SOD), peroxidase (POD), catalase (CAT), and ascorbate peroxidases (APX). SOD catalyzes the dismutation of O_2_^∙−^ into H_2_O_2_ and O_2_, whereas APX uses ascorbate as an electron donor to reduce H_2_O_2_ to water. CAT also detoxifies H_2_O_2_ by converting it into water and oxygen. The ascorbate–glutathione (AsA-GSH) cycle also contributes to maintaining the cell redox state [97]. Tolerant rice cultivars consistently produce higher activities of antioxidant enzymes, which may assist to maintain lower levels of H_2_O_2_ and malondialdehyde (MDA) [98,99,100]. The non-enzymatic system, where plants neutralize or alleviate damage to harmful substances by producing osmotic regulators, such as ascorbate, glutathione, and polyphenolics etc. [101]. The non-enzymatic antioxidant system comprises low-molecular-weight compounds that alleviate oxidative damage in cells and tissues via direct neutralization of free radicals and ROS. The presence of compatible solutes-for example, proline and glycine betaine (which also act as ROS scavengers)-confers protection on macromolecules and the photosynthetic apparatus [102]. Correspondingly, transcript levels of antioxidant genes (*OsAPX2, OsCATA, OsGR*) are markedly upregulated under stress in tolerant genotypes [103]. These representative ROS-scavenging genes are summarized in Table 2.

However, higher ROS accumulation is harmful; controlled ROS production is a vital secondary messenger to initiate defense cascades [104]. Tolerant plants fine-tune this balance, leveraging ROS signaling while preventing cytotoxicity. For example, the receptor-like kinase *OsSTRK1* phosphorylates and activates CAT, maintaining ROS homeostasis and reducing root cell death under salinity [105]. Similarly, transcription factors SNAC1 and OsSKIPa genes enhance tolerance by stomatal regulation and enhanced antioxidant capacity [106,107]. Under alkaline conditions, rapid ROS production is generated due to disrupted electron transport; tolerant genotypes defend the ROS damage by elevated enzymatic activity and root-oxidizing ability [108]. A major unresolved issue is how to separate protective ROS signaling from uncontrolled oxidative damage. Tolerant genotypes often show a transient ROS burst followed by strong antioxidant induction, whereas sensitive genotypes exhibit sustained ROS accumulation. Yet attempts to improve tolerance by overexpressing a single antioxidant enzyme have produced inconsistent results in the field, indicating that the causal relationship between antioxidant capacity and tolerance is more complex than originally assumed. We therefore classify enhanced antioxidant activity as a moderately supported mechanism that requires confirmation through multi-gene manipulation or field-validated markers.

### 3.5. Effects on Photosynthesis and Metabolism

Saline–alkaline stress seriously constrains rice productivity by impairing photosynthetic efficiency and carbon–nitrogen metabolism. The limiting factors modify with the duration and severity of stress. During the initial stage of stress, photosynthesis is generally dominated by stomatal and hydraulic limitations, as reduced water uptake lowers leaf water status and stomatal closure restricts CO_2_ diffusion [40,43]. Both CO_2_ assimilation is limited and the stomatal is closed at the begging of the osmotic stress [40]. Under prolonged or severe stress, mesophyll, photochemical, and biochemical limitations may become increasingly important. Simultaneously, toxic Na^+^ and Cl^−^ accumulate in leaves which cause the degradation of chlorophyll and destruction of chloroplast ultrastructure [45,109]. Damaging the oxygen-evolving complex and dissociation of peripheral proteins inhibit electron transport [110]. Therefore, the net photosynthetic rate (An) is reduced by more than 50% at moderate salinity (75 mM NaCl) [40]. Tolerant genotypes retain higher chlorophyll content, stomatal aperture and mesophyll tolerance, which is able to sustain the function of Calvin cycle enzymes (Rubisco), and to continue carbon assimilation [111]. Thus, early tolerance relies on water status and stomatal regulation, whereas tolerance under prolonged or severe stress depends on protecting chloroplasts, electron transport and Calvin cycle function. The transition from stomatal to non-stomatal limitation remains poorly defined, particularly under fluctuating field conditions. Conflicting reports on the relative importance of stomatal versus biochemical limitation may result from differences in stress severity, exposure duration, and measurement techniques. Moreover, the common assumption that stress tolerance inevitably trades off with growth is not universal: some wild rice accessions maintain high photosynthesis under salinity, suggesting that tolerance costs may be lower in certain genetic backgrounds than in modern cultivars.

At the metabolic level, the cell will allocate more energy to maintain life but not to grow. Limited photosynthate is used for stress response, such as osmoregulation (proline, sugars) and ion pumping [43,112]. During the grain-filling stage, salinity suppresses key starch biosynthetic enzymes such as sucrose synthase and ADP-glucose pyrophosphorylase, leading grain chalkiness and reduced amylose content [113]. Furthermore, proteomic analyses revealed that simultaneous upregulation storage protein synthesis (glutelins) and downregulation starch metabolism led to an altered grain protein–starch ratio under saline–alkaline stress [114,115]. The accumulation of sugar in the leaves under stress conditions is attributed to the metabolic adaptation and feedback inhibition of photosynthesis [40,116]. Tolerant varieties have relatively strong sinks and can still transport assimilate to the grains.

Alkaline stress primarily induces micronutrient deficiencies. Fe and Zn are usually deficient under high pH conditions, causing chlorosis and low-leaf areas and decreasing photosynthetic capacity [117]. However, tolerant genotypes can produce more organic acids (citrate, malate) to solubilize rhizosphere Fe^3+^, thereby preserving chlorophyll biosynthesis [118]. Beyond organic acid exudation, alkalinity-tolerant rice can upregulate Fe-deficiency-responsive genes such as *OsIRT1*, *OsYSL2*, and *OsNAS* family genes, and Zn transporters such as OsZIP family members, to enhance Fe/Zn acquisition and trans-location under high pH [117,118,119]. Additionally, the transcription factor *OsMADS25* and the G-protein γ subunit GS3/AT1 have been proposed as alkaline-tolerance candidates on the basis of root-growth regulation and ROS homeostasis [120,121]; both require functional validation before being treated as established alkalinity determinants. Stress can also damage the source-sink system, reduce the number of spikelets (sinks) before anthesis, damage the function of flag leaves (sources) and stem reserve assimilate after anthesis. Tolerant cultivars have high translocation efficiency of stem NSC to partially compensate for photosynthetic inhibition [82,122]. Saline–alkaline tolerance is defined as the ability to keep photosynthesis rates, resource allocation and sink activity to ensure yield stability. Alkali stress depresses photosynthesis mainly through micronutrient-deficiency-induced chlorosis rather than direct ion toxicity, whereas salinity-induced photosynthetic decline is dominated by osmotic closure and Na^+^/Cl^−^ accumulation. This contrast again emphasizes that the three stress types converge on yield loss through different physiological routes.

**Table 2:** Major genes and quantitative trait loci (QTLs) associated with saline–alkaline stress tolerance and grain quality in rice.

Gene/QTL (Chromosome)	Function/Associated Trait	Notes on Contribution to Tolerance or Quality	References
*OsHKT1;5* (Saltol QTL, chr 1)	Na^+^ transporter (uniporter) for Na^+^ retrieval from xylem (root)	Limits Na^+^ loading into shoots; maintains low leaf Na^+^ and high K^+^: Na^+^ ratio. Allele from Pokkali (Saltol QTL) confers seedling-stage salt tolerance.	[18]
*OsSOS1* (chr 12)	Plasma membrane Na^+^/H^+^ antiporter (root, shoot)	Pumps Na^+^ out of cells, critical for root Na^+^ extrusion and maintaining low cytosolic Na^+^. Mutants are salt-hypersensitive; overexpression improves tolerance.	[19,20]
*OsNHX1* (chr 7)	Vacuolar Na^+^/H^+^ antiporter	Sequesters Na^+^ into vacuoles in shoot and root cells, reducing cytosolic Na^+^ toxicity. Transgenic overexpression increases salt tolerance (better growth and ion balance under salt).	[21,22]
*SKC1* (chr 1)	QTL for Shoot K^+^ Content (encodes OsHKT1;5)	Same locus as HKT1;5 above. Identified in salt-tolerant landrace Nona Bokra; helps retain K^+^ in shoots under salt by lowering Na^+^ influx. Improves growth and reduces leaf Na^+^.	[18]
*OsAKT1* (chr 6)	Inward-rectifying K^+^ channel (root uptake)	Maintains K^+^ uptake under low external K^+^ and in presence of Na^+^. Tolerance linked to keeping higher K^+^ uptake. Requires OsCBL1/OsCIPK23 for activity; salt stress can inhibit AKT1, but tolerant plants mitigate this.	[23,24]
Deeper Rooting 1 (*DRO1*) (chr 9)	Root growth angle determinant	Not a salt-specific gene but confers deeper rooting. Can help plants avoid surface salt by acquiring water from deeper soil. Near-isogenic lines with deeper roots showed better salt avoidance in some studies.	[25,26]
*OsCIPK24* (OsSOS2) (chr 1)	Calcineurin B-like protein-interacting kinase	Central in SOS pathway: activated by Ca^2+^ via OsCBL4 (SOS3); phosphorylates OsSOS1 and possibly OsNHX1 to activate them under salt stress. Essential for ion homeostasis signaling.	[46]
*OsCBL4* (OsSOS3) (chr 4)	Calcium sensor for salt stress (CBL family)	Perceives salt-induced Ca^2+^ spikes; forms complex with CIPK24/SOS2 to trigger downstream antiporters. Mutations cause salt hypersensitivity.	[30]
*OsAPX1*/*OsCATC* (various)	Ascorbate peroxidase/Catalase (antioxidant enzymes)	Scavenge ROS (H_2_O_2_) in chloroplasts and peroxisomes. Higher expression/activity in tolerant cultivars limits oxidative damage under salt. QTLs for salt tolerance often coincide with loci boosting antioxidant enzyme levels.	[33,34]
*STRK1* (chr 4)	Salt Tolerance Receptor-like Kinase	Enhances ROS scavenging by phosphorylating OsCATC (a catalase), thus improving H_2_O_2_ detoxification. Overexpression of *STRK1* increases salt tolerance (better root growth, less oxidative damage).	[29]
*SNAC1* (chr 12)	Stress-responsive NAC transcription factor	Confers drought and salt tolerance when overexpressed by regulating stomatal closure (ABA signaling) and enhancing expression of LEA proteins and antioxidants. Improves survival under salt and yields in field tests (minimal yield penalty).	[35]
*OsbZIP23* (chr 2)	bZIP transcription factor (ABA-responsive)	Master regulator of many stress-inducible genes (osmolyte synthesis and oxidative stress genes). Overexpression leads to improved salt tolerance via enhanced ABA sensitivity.	[36]
*OsDREB2A* (chr 1)	DREB transcription factor (ABA-independent)	Regulates a suite of osmotic/drought-responsive genes. A constitutively active form in rice improved tolerance to salt by upregulating LEAs, dehydrins, etc. However, overexpression can stunt growth (must be carefully regulated).	[40,123]
*BADH2* (chr 8)	Betaine aldehyde dehydrogenase (enzyme in glycine betaine synthesis)	Functional BADH2 is required for glycine betaine production (osmoprotectant). However, cultivated aromatic rice carry a non-functional badh2 (leading to aroma but no betaine). Functional BADH2 can help osmotic stress tolerance by enabling some betaine accumulation. Overexpression of a BADH gene from other sources increases salt tolerance.	[37,38]
*P5CS* (chr 10)	Δ^1^-pyrroline-5-carboxylate synthase (enzyme in proline biosynthesis)	Key regulatory enzyme for proline synthesis. Upregulated by salt via osmotic stress signaling. Transgenic rice overexpressing *P5CS* accumulates more proline and shows improved salt tolerance (better growth and water status).	[39]
*OsSAP16* (chr 1)	Stress-Associated Protein (zinc-finger family)	SAP genes act as regulators of stress responses. OsSAP16 overexpression enhanced both salt and drought tolerance, possibly by modulating ionic and osmotic stress signaling. SAP16 as a transcriptional regulator of defense genes.	[30,41]
QTL qSaltol (chr 1)	Major QTL for seedling-stage salt tolerance (specific to Pokkali-derived mapping populations)	Associated with low Na^+^ uptake and better survival in seedling stage. Explains 40% of variation in shoot Na^+^/K^+^ ratio under salt. HKT1;5 is the known candidate gene here, but other nearby genes (OsCPK17, a Ca^2+^-dependent kinase) might also contribute. Marker-assisted introgression of qSaltol into popular varieties improved their seedling salt tolerance.	[13,42]
QTL Saltol2 (reproductive) (on chr 4, 7)	QTLs for salt tolerance at flowering/reproductive stage	Several QTLs identified for tolerance during flowering (maintaining grain set). One on chr 7 (from Nona Bokra) linked to spikelet fertility under salt, another on chr 4. These may involve genes like OsHKT1;1 (chr 6) or others affecting pollen development under stress.	[42,43]
*OsMADS25* (chr 4)	MADS-box transcription factor	Upregulated under high-pH stress; candidate regulator of root adaptation. Evidence status: correlative (expression data); no loss-of-function confirmation in rice.	[32]
*Wx* (GBSSI) (chr 6)	Granule-bound starch synthase (amylose synthesis in endosperm)	Major gene for rice grain amylose content (and thus eating quality). Salt stress during grain fill tends to downregulate *Wx* expression, leading to lower amylose and chalkier texture. Maintaining *Wx* expression under stress is important for grain quality, tolerant varieties or transgenics that keep GBSSI active have better grain filling under salt. *Wx* alleles also interact with environment: some high-quality low-amylose varieties suffer yield penalty under salt due to altered starch deposition.	[45,77]
*Chalk5* (chr 5)	Vacuolar H^+^-translocating pyrophosphatase (VPP)	Chalk5 is a gene controlling grain chalkiness; a mutation in a VPP enzyme leads to higher chalkiness in rice. Under stress, altered vacuolar ion balance can affect starch packing. Varieties with functional Chalk5 (lower chalkiness) may handle stress grain filling better. There is evidence salt stress upregulates some VPPs (perhaps to sequester Na^+^ in amyloplasts) but the relationship to chalkiness is complex. Nonetheless, Chalk5 is a target for improving grain appearance under stress conditions.	[124,125]
*BADH2 (aroma)* (chr 8)	Betaine aldehyde dehydrogenase 2—quality trait gene (aromatic vs. non-aromatic rice)	Non-functional alleles cause aroma (loss of function leads to 2-acetyl-1-pyrroline accumulation). Aromatic varieties (Basmati, Jasmine) thus cannot synthesize glycine betaine. Functional BADH2 is required for glycine betaine synthesis. Aromatic varieties carrying non-functional badh2 cannot accumulate betaine, which may reduce osmoprotective capacity; however, a consistent penalty in field salinity tolerance has not been demonstrated, and aroma–tolerance trade-offs should be treated as hypothetical. Evidence status: transgenic/controlled-environment.	[31,123]
*SSIIa (ALK)* (chr 6)	Starch synthase IIa-Quality gene (gelatinization temperature)	Allelic differences in SSIIa (Alk gene) determine rice grain gelatinization temperature and texture. Stress during grain filling can disrupt SSIIa activity, affecting amylopectin structure. Varieties with certain SSIIa alleles yield softer or harder rice; under salt stress, SSIIa expression might be altered, influencing final eating quality. Maintaining proper SSIIa function under stress is important to avoid drastic changes in cooking quality. For coordinated improvement, breeders consider SSIIa alleles so that salt-tolerant lines still meet quality preferences.	[116,126]
*AT1/GS3* (chr 3 in sorghum; rice homolog *GS3* on chr 3)	G-protein γ subunit—controls grain size in rice; identified as Alkaline Tolerance 1 (AT1) in sorghum (for alkaline pH tolerance)	G-protein γ subunit; major grain-length gene in rice; AT1 orthologue confers alkaline-pH tolerance in sorghum. Rice gs3 mutants show longer grains; enhanced alkaline tolerance has been reported but is inferred mainly from sorghum and awaits confirmation in rice field trials. Evidence status: indirect/cross-species.	[48]
Susy/AGPase	Sucrose synthase and ADP-glucose pyrophosphorylase; starch biosynthesis	Downregulated during grain filling under salinity, limiting starch deposition and grain weight.	[113,114]
Glutelin/Prolamin genes	Storage protein synthesis	Upregulated under salt stress, increasing protein-to-starch ratio and lowering eating quality.	[114,115]

Note: This table includes genes for stress tolerance (upper rows) and grain quality (lower rows) given the review’s focus on coordinating yield and quality. Many additional genes/QTLs influence salinity tolerance (e.g., OsHKT2;1/2;4 for Na^+^/K^+^ transport, OsTPP1 for trehalose, OsNAC6, OsCPK17, etc.) and grain quality under stress (e.g., OsGAD for GABA accumulation affecting taste). The listed entries are those frequently reported or of high significance in recent literature. QTL names are in quotes if not a single cloned gene.

### 3.6. Molecular Basis of Grain Quality Alteration Under Saline–Alkaline Stress

Saline–alkaline stress-induced grain quality loss is not only a downstream consequence of limitation of assimilate supply, but also a coordinated reprogramming of protein metabolism storage and starch in growing endosperm [127]. Starch synthesis in rice endosperm occurs via a cascade of starch-synthesizing enzymes, including sucrose synthase, ADP-glucose pyrophosphorylase (AGPase), granule-bound starch synthase I (GBSSI/Wx), soluble starch synthases (SSI, SSIIa, SSIIIa), starch-branching enzyme (SBEI, SBEIIb) and starch-debranching enzyme (ISA1, PUL) [127]. Saline–alkaline stress downregulates many of these genes, with Wx and SSIIa being especially sensitive, leading to decreased amylose synthesis and altering the amylopectin chain length distribution [128]. The consequences of an increase in short-chain amylopectin and decrease in long-chain fractions are linked with higher gelatinization temperature and deteriorated cooking and eating. Under salt stress, free space between starch granules and the remaining air space within the endosperm result in chalkiness formation in the endosperm. This is associated with the downregulation of Chalk5, a vacuolar H^+^-translocating pyrophosphatase, and other chalkiness-associated loci, which disrupt endomembrane pH homeostasis and the orderly deposition of storage substances [129]. At the same time, under stress, the leaves begin to yellow and fall off, and the grain-filling period is shortened, which lowers the rate and duration of starch deposition, resulting in loosely packed, chalky grains with lower 1000-grain weight and head-rice yield [1,116,126]. Storage protein accumulation shows an opposite trend. Salt stress upregulates glutelin and prolamin synthesis and nitrogen remobilization from senescing leaves, increasing grain protein content and the protein-to-starch ratio [114,115]. This change together with the low amylose content also results in harder texture, low palatability and poor milling quality. At the molecular level, the antagonistic regulation of starch and protein biosynthesis appears to be mediated by sugar- and nitrogen-signaling pathways, including SnRK1, TOR, and bZIP transcription factors, although their specific roles under saline–alkaline stress remain poorly defined [114,130]. Therefore, the deterioration in grain quality caused by saline–alkaline stress is due to a shift in the endosperm carbon–nitrogen allocation, as a result of stress, and not from a single gene defect. This requires coordinated expression of Wx, SSIIa, Chalk5 and storage protein genes, as well as proper assimilate supply during the grain filling stage, to ensure yield and quality.

## 4. Agronomic Interventions for Managing Saline–Alkaline Stress

**Figure 3:**
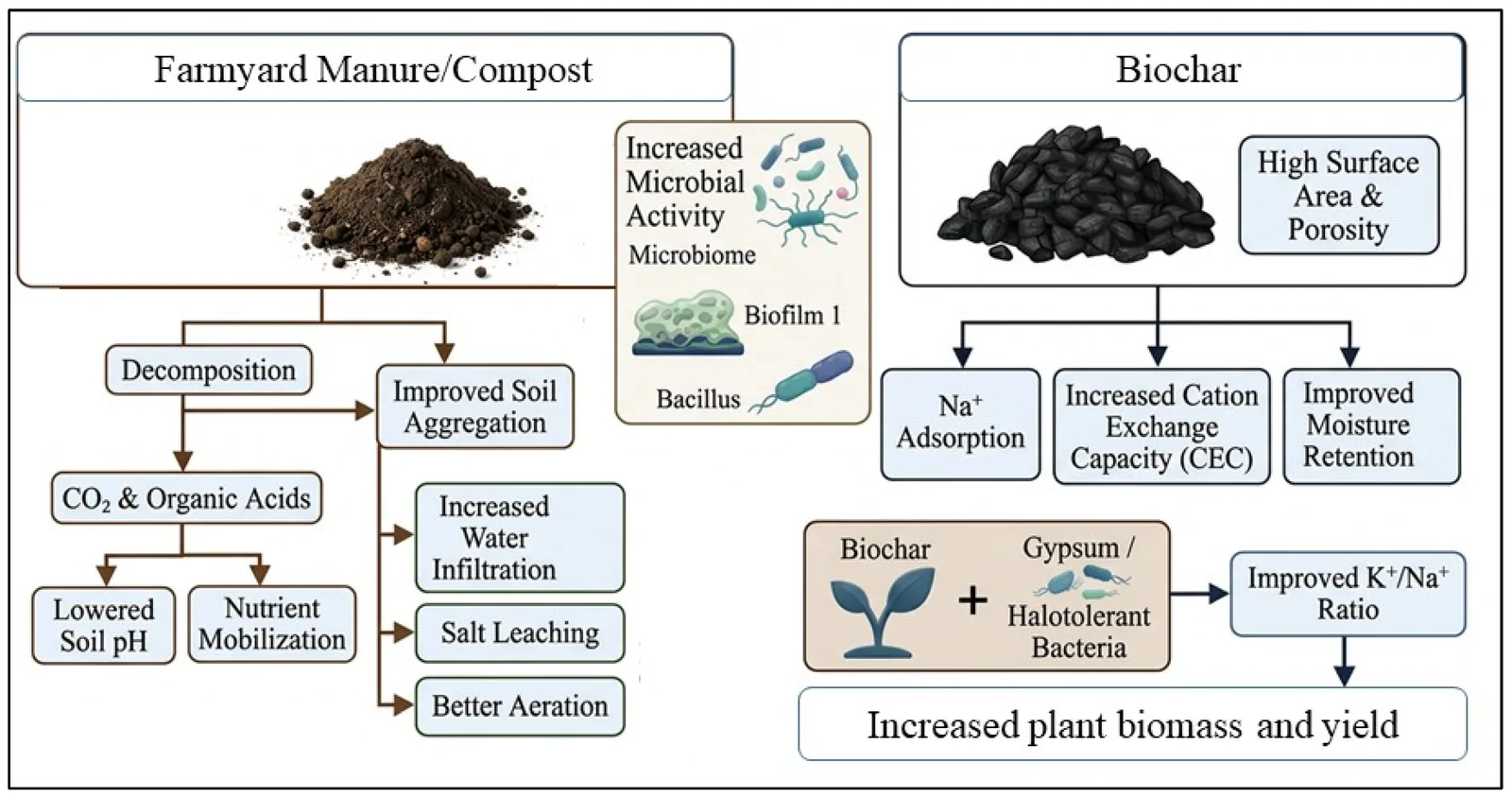
The saline–alkali soil improvement using organic amendments and related materials. Farmyard manure/compost enhances microbial activity (microbiome, biofilm formation), improves soil aggregation, and promotes decomposition, producing CO_2_ and organic acids that lower soil pH. These changes facilitate nutrient mobilization, increased water infiltration, salt leaching, and better aeration, ultimately boosting plant biomass and yield. Biochar contributes through Na^+^ adsorption, increased cation exchange capacity, improved K^+^/Na^+^ ratio, enhanced microstructure and porosity, high surface area, and better water holding capacity and retention, leading to improved soil structure and water dynamics. Gypsum and halotolerant bacteria also play supportive roles in this remediation process.

Cultivation practices play a vital role in crop production under saline–alkaline condition, almost contributing equally to yield as breeding [131]. In salt-affected soils, integrated soil, water, fertilizer, and crop management methods can effectively restore rice productivity and grain quality. This section provides a brief overview of the key cultural management strategies and their underlying mechanisms [27,132] (Table 3, Figure 3).

### 4.1. Soil Reclamation and Amendments

#### 4.1.1. Leaching and Drainage

Leaching combined with drainage is the main strategy for improvement saline soil, whereby normal irrigation water is used to wash excess salts below the root zone under drainage conditions [133]. Before transplanting, paddy fields are usually flooded to dissolve and eliminate salts, and subsequently drained to export solute salts. This method is effective for reducing salinity, but it relies on complete drainage infrastructure to prevent groundwater levels rising and subsequent salt accumulation in the surface soil again. In irrigated regions, subsurface drainage pipes or deepened ditches reduce groundwater levels to inhibit salt accumulation [134]. However, in coastal areas, the method of tidal water exchange irrigation is used to achieve gradual salt depletion. It was reported that an increase of 15–20% leaching above crop water demand can significantly lower soil salinity and improve rice tolerance [135]. Nevertheless, leaching barely improves sodic or alkaline soils without amendments such as gypsum. Only remove of soluble salts leaves Na^+^ retained on soil cation exchange sites and maintains elevated soil pH [27].

#### 4.1.2. Chemical Amendments

Improvement of sodic (alkaline) soils usually require chemical amendments. Gypsum (CaSO_4_·2H_2_O) is the most widely applied amendment, which can supply calcium source to displace Na^+^ from soil cation exchange sites. Its primary mechanism involves Ca^2+^ supply, displacement of Na^+^ from soil exchange sites, and subsequent removal of Na^+^ through leaching, which lowers ESP and improves soil aggregation and water infiltration [27,28]. The displaced Na^+^ is subsequently leached out as soluble sodium sulfate (Na_2_SO_4_) by irrigation or rainfall [28]. Gypsum is a neutral salt, so it does not acidify the soil directly. Its effect on pH is indirect: Ca^2+^ reacts with carbonate and bicarbonate to precipitate poorly soluble CaCO_3_ (Na_2_CO_3_ + CaSO_4_ → CaCO_3_ + Na_2_SO_4_), which removes alkalinity, and the lower ESP reduces N^+^-driven alkalinity. The pH decrease is therefore usually moderate [27]. It has been demonstrated that gypsum application to sodic soils in rice field significantly decrease soil pH and exchangeable sodium percentage (ESP), and remarkably increase grain yield [136]. For moderately sodic soils, the recommended gypsum rate is approximately 5–10 t ha^−1^ [137]. Flue-gas desulfurization gypsum (FGD), a by-product of coal-fired power plants, applied at 30% of the conventional rate to sodic soil reduced pH from 9.4 to 8.3 and enhanced rice yield within a few years [138]. Other amendments include elemental sulfur and acid-forming materials (sulfuric acid, iron sulfate), can generate sulfuric acid in situ. This acid dissolves native calcium carbonate and neutralizes soil alkalinity [27]. Unlike gypsum, these materials lower pH directly by generating acid. In some systems, organic acids or acid-producing organic amendments (e.g., pyrite, ferrous sulfate) are used to reduce pH. Notably, mixed application ferrous sulfate with organic manure enhances rice yield in sodic soils by supplying available iron and lowering soil pH [139].

#### 4.1.3. Organic Amendments

Including farmyard manure, compost, crop straw, green manure, biochar, and humic substances, play a vital role in reclaiming saline–alkali soils. Their functions include improving soil structure, enhancing water infiltration, providing slow-release nutrients, and releasing humic substances that can chelate sodium ions (Na^+^) or deliver bioavailable calcium (Ca^2+^) [140,141,142]. Conventional organic amendments (such as farmyard manure and compost) can enhance soil aggregation, promote salt leaching and improve soil aeration in sodic soils. Meanwhile, organic decomposition releases CO_2_ and low-molecular-weight organic acids, which moderately reduce soil pH and enhance nutrient mobilization [143]. A five-year field experiment demonstrated that annual manure application at 20–30 t ha^−1^ lowered soil pH by 0.3–0.5 units and increased rice grain yield by 15–25% (Figure 3). Biochar is a charcoal-like carbonized biomass with a large specific surface area that enhances porosity and cation exchange capacity. In saline soils, biochar adsorbs Na^+^ and soluble salts, thereby mitigating their bioavailability to crops [144]. In saline soils, biochar application alleviates salt stress in plants by enhancing soil water retention and providing favorable microhabitats for soil microorganisms [145]. The interaction of biochar with gypsum or halotolerant plant growth-promoting bacteria generates notable synergistic effects [140,146]. One controlled study revealed that application of 10 t ha^−1^ biochar mixed with a salt-tolerant bacterial inoculant significantly increased rice biomass and the shoot K^+^/Na^+^ ratio compared to single amendments (Figure 3). The integrated soil amelioration strategy is summarized in Table 3.

**Table 3:** Agronomic management strategies to mitigate saline–alkaline stress in rice, with their modes of action and reported benefits.

Intervention	Mechanism/Action	Reported Benefits in Rice Systems (Examples)	References
Leaching and Drainage	Flushes soluble salts out of root zone; requires good drainage (e.g., via channels or tile drains).	Reduces soil salinity (EC) before cropping; prevents salt accumulation over time. In coastal Bangladesh, dry-season flushing of fields with fresh water-cut soil ECe from 8 to 3 dS/m, enabling a rice crop where previously none grew. Installing drains in Pakistan’s salt-affected polders improved rice yield by 25–40% by lowering the water table and salinity [147].	[18,19]
Gypsum application	Calcium source replaces Na^+^ on exchange sites; forms Na_2_SO_4_ which leaches; lowers pH as Ca^2+^ + 2Na^+^ exchange flocculates clays.	Lowers soil ESP and pH, improving soil structure and infiltration. Northeast China: 30% FGD gypsum mixed in topsoil reduced pH by 1 unit and enabled rice cultivation with yields 4 t/ha on formerly barren sodic land.	[20]
Organic amendments (manure, compost, straw)	Improve soil structure (aggregation), increase water holding, add Ca^2+^, and produce CO_2_/organic acids upon decomposition (slight pH reduction, micronutrient chelation).	Add nutrients (N, P, etc.) and enhance microbial activity. Farmyard manure (FYM) 10 t/ha in saline soil lowered ECe by increasing percolation and improved rice tillering and biomass. Continuous rice-straw incorporation in sodic soil raised soil organic C and lowered pH by 0.2, resulting in 15% higher yield vs. no amendment. Manure (5 t/ha) plus gypsum outperformed either alone: in a sodic field, combo gave 20% more yield than gypsum only.	[21,22,23]
Biochar addition	Highly stable carbon improves CEC and water retention; can adsorb Na^+^ and other ions; often raises soil organic carbon significantly.	In a coastal saline paddy, rice yield increased 10–15% with 5–10 t/ha rice-hull biochar, mainly due to improved soil moisture and nutrient retention. Biochar 10 t/ha in a sodic soil decreased exchangeable Na by 5% and pH by 0.3 (due to enhanced leaching), aiding rice root growth. When combined with salt-tolerant PGPR, biochar further improved K^+^/Na^+^ ratios and shoot biomass under salinity.	[24,25,26]
Irrigation management (flooding, freshwater use, cyclic use)	Maintains water cover to dilute salts (rice can tolerate higher water salinity than soil salinity when flooded); scheduling to avoid stress at sensitive stages; blending or alternating saline and non-saline water.	Continuous shallow flooding (2–5 cm) in salt-affected fields prevents salt concentration at surface; e.g., in Egypt, rice flooded throughout growth had 30% higher yield than intermittently irrigated rice under the same saline water (EC 4 dS/m). Alternate use of canal (fresh) and tubewell (saline) water can keep average salinity manageable: farmers in NW India apply one fresh irrigation before panicle initiation to ensure low salt during flowering, which preserved 80% of yield potential if saline water was used exclusively. Early-season drainage of floodwater with accumulated salts (from capillary rise) and refreshing with rain or fresh water significantly improved seedling survival in coastal polders.	[46]
Field layout and mulching (land leveling, mulches)	Precise leveling ensures uniform water coverage (avoids salt concentrating in high spots); mulching (straw, plastic) reduces evaporation and salt crusting; furrow vs. bed planting to manage salt distribution.	Laser-leveled salt-affected fields had 10% higher rice yield than unleveled, as uniform flooding prevented dry patches and salt accumulation. Rice straw mulch (5 cm thick) during fallow reduced soil evaporation by 30% and lowered surface salinity (EC 0–10 cm) by 15%, leading to better seedling establishment next season. In heavy saline soils, placing seeds on slightly raised beds with irrigation in furrows pushes salt into furrows and away from seed zone; this method improved stand establishment and yield compared to flat planting in a trial on ECe 15 dS/m soil.	[29,33,34]
Nutrient management (K, Zn, N, etc.)	Supplemental fertilizers to counteract nutrient imbalances: extra K to compete with Na^+^; Zn and Fe to alleviate deficiency in high pH; N and P management to support growth under stress; timing adjustments.	Potassium: Additional K fertilizer in saline soil improves rice K^+^ nutrition and reduces Na^+^ uptake. E.g., applying 25% more K than standard recommended dose increased grain yield by 10% under salinity (ECe 6), and improved K: Na ratio in shoots. The mechanism is mass-action and K^+^ uptake enhancement, which helps osmotic balance and enzyme function. Zinc: Foliar spray or soil application of zinc sulfate in alkali soils corrects Zn deficiency and boosts grain yield and quality. In sodic fields (pH 9), 5 kg/ha ZnSO_4_ each season raised rice yields by 15% and reduced empty grain percent. Nitrogen: Split application of N (more at later stages) under salinity can compensate for stress-induced N losses/inefficiencies. Also, use of Ammonium-N (NH_4_^+^) is preferable in high pH soils (helps acidify rhizosphere). Silicon: Although not a classic nutrient in many regimes, silicate fertilizer (like calcium silicate) has been shown to mitigate salinity effects by strengthening cell walls and reducing Na uptake. Trials in Vietnam showed adding Si (120 kg/ha) improved salt-stressed rice yield by 8–10% and reduced leaf Na.	[35,36,123]
Microbial inoculants (PGPR, arbuscular mycorrhiza)	Introduction of salt-tolerant plant growth-promoting rhizobacteria (PGPR) or fungi that improve nutrient uptake, produce growth hormones, ACC deaminase (lowers ethylene), or help in osmotic adjustment; some solubilize bound nutrients in alkali soils.	PGPR: For example, *Pseudomonas stutzeri* and *Bacillus safensis* strains from coastal soils, when inoculated to rice, increased grain yield by 10–20% under EC 8 dS/m by enhancing root growth and K^+^ uptake. These bacteria produced ACC deaminase (reducing ethylene stress) and exopolysaccharides that help soil aggregation and moisture retention. Azolla and BGA: In alkaline sodic paddies, dual cropping with Azolla (an aquatic fern with N-fixing cyanobacteria) improved rice yield by adding N and organic matter and possibly releasing organic acids that lower pH. Trials in India showed 0.5 t/ha yield increase with Azolla incorporation vs. no Azolla on pH 9 soil. Mycorrhiza: Although paddy conditions are not always conducive for mycorrhizae, some salt-tolerant AM fungi can colonize rice roots under upland or alternate wetting conditions. Inoculation with *Glomus* intraradices in pot experiments enhanced rice P uptake and yield under salt stress. The fungi likely helped in nutrient uptake and imparted some hormonal signals for stress tolerance. Consortia: Combined biofertilizer consortia (including *Azospirillum*, *Bacillus*, *Rhizobium*, etc.) on saline soils have yielded notable improvements. For example, in Egypt, a consortium increased rice grain yield by 18% under EC 5 dS/m compared to uninoculated.	[37,38,39,40]

### 4.2. Integrated Management

In the improvement of saline–alkaline land, the combined application of amendments is significantly more effective than using them individually. Field evidence illustrates that the combined amendment of desulfurization gypsum (DG), sandy loam, and farmyard manure (FM) increased rice yields by 24% more than single application. The combination treatment elevated the soil osmotic potential and improved K^+^/Na^+^ selectivity in rice tissues, facilitating superior potassium uptake [148,149]. The International Rice Research Institute (IRRI) recommends a multi-component strategy for coastal saline soils, including salt-tolerant varieties, optimized planting dates, targeted amendments, land leveling, salt flushing, increased fertilization (especially K and Zn), and stage-specific water management [150]. Bangladesh and India have enhanced the production of saline soil by extending the dry-season cultivation period to add another harvest [151]. Moreover, the amendment to practices applied during grain filling can also improve grain quality. For example, sufficient K nutrition can increase grain yield and improve the size and quality of grains by ensuring proper carbohydrate translocation (since K is essential for phloem transport). Zinc nutrition can improve grain micronutrient content and potentially reduce chalkiness and unfilled grains in alkali soils [30]. Collectively, these practices are central to the coordinated regulation of yield and quality by alleviating stress.

## 5. Breeding Strategies and Genetic Improvement

Rice breeding for saline–alkali stress is an important approach to improving the yield and quality of saline–alkali land. During the breeding progress, traditional varieties (Pokkali, CSR series) laid the groundwork, but the yield preformation is too low [122]. Modern pipelines combine landrace hybridization, MAS, genomic selection, and gene editing, incorporating quality traits (amylose, grain morphology) to meet market demands [152]. key donors, QTL/gene mapping, trait pyramiding, and biotechnological advances were reviewed in this section (Table 2).

### 5.1. Donor Germplasm and Conventional Breeding

Historically, elite landraces with intrinsic tolerance have served as the foundation for salt-resistant rice breeding. Among them, Pokkali (India) and Nona Bokra (Bangladesh/India) remain pivotal donor parents. Pokkali donor is the source for Saltol QTL on chromosome 1 while Nona Bokra donor is the source for the *SKC1* QTL which was cloned as *OsHKT1;5*. These are not independent genes, but rather *SKC1* is a QTL name, *OsHKT1;5* is the cloned gene and Saltol is a QTL interval in which *OsHKT1;5* is the major candidate gene. Thus, Pokkali and Nona Bokra represent different donor sources and alleles at the *OsHKT1;5* locus, together with additional tolerance loci [16,56,153,154]. However, these original parents exhibit tall plant height, low yield, and poor grain quality. Through extensive cross-breeding, these quantitative trait loci (QTLs) have been introgressed into high-yielding genetic backgrounds, leading to improved lines such as IR63307-4B-4-3 [132]. In addition, utilizing wild relatives provides extra genetic resources. For example, *Porteresia coarctata* (*syn.* *Oryza coarctata*) grow well in mangrove ecosystems and supply candidate genes (e.g., vacuolar H^+^-ATPase subunits) for ion sequestration [58].

Conventional selection has led to the release of commercial varieties worldwide, including India’s CSR series (tolerant to pH 9.2 or ECe 6–8 dS m^−1^), Bangladesh’s BRRI dhan series, and China’s “Sea Rice” cultivars (e.g., Haiyuan 23 and Searice 86) [152,155,156]. In the Chinese breeding programs, these cultivars were developed from coastal saline–alkali-tolerant breeding materials and wild rice relatives collected from mangrove-adjacent intertidal wetlands. We use the term “mangrove-adapted germplasm” in this restricted sense: it refers to rice or wild-rice accessions originating from mangrove-adjacent coastal habitats that display tolerance to salinity, waterlogging and high pH. It does not imply that the cultivars descend from mangrove plants, nor that mangrove species were used as donors in their pedigrees. These varieties contribute to food security on marginal lands, with reported yields of 3–5 t ha^−1^ under favorable management, although their grain quality has historically been inferior.

### 5.2. Marker-Assisted Selection (MAS) and QTL Pyramiding

The use of major QTLs, particularly the *Saltol* QTL on chromosome 1, has significantly advanced MAS-based breeding. The most famous line is FL478. It is an introgression of the Saltol locus containing the *HKT1*;5 loci from Pokkali into IR29, and is a global donor parent [153]. MAS has also encouraged the use of Saltol in elite varieties like BRRI dhan28-Saltol and dhan29-Saltol in Bangladesh. In multi-location trials, these lines showed improved seedling survival and growth under salt stress, with yield and grain quality comparable to the recurrent parents [157]; performance under farmer’s field conditions has been less extensively documented. Likewise, the IR64-*Saltol* derivative in the Philippines is more field-tolerant [158].

In addition to Saltol, MAS also attempts to use other complementary loci to obtain stage-specific tolerance. *OsHKT1;1* (Os04g51830) is located on chromosome 4, not chromosome 8 or chromosome 6, and encodes a Na^+^ transporter associated with root Na+ content and ion homeostasis [159]. A reproductive-stage salt-tolerance QTL from Nona Bokra has been mapped to chromosome 7, and another to chromosome 4, but *OsHKT1;1* is not located within those intervals; candidate genes for these QTLs remain to be confirmed [16,154]. The present strategies focus on QTL pyramiding. This approach involves the incorporation of tolerance at the seedling stage (Saltol), reproductive stage tolerance, and ion-homeostasis traits (e.g., *qSKC1* for K^+^ homeostasis). Although epistatic interactions and minor-effect QTLs bring challenges, multi-stress resilient lines have been developed. For example, some lines integrate the traits of Saltol, SKC1 and Sub1 (submergence tolerance) to adapt to altered coastlines [160]. For sodic soils (high-alkaline pH), the recent discovery of the *GS3/AT1* gene offers a new MAS target. These shift breeding from indirect selection for pH resilience to direct genetic changes.

MAS also contributes to the recovery of high-quality traits in tolerant backgrounds. By utilizing the diagnostic markers for Wx (amylose), ALK/SSIIa (gelatinization temperature), GBSS (grain quality) and BADH2 (aroma), breeders can monitor complex-quality alleles. An example of this is the development of Basmati rice that is salt-tolerant. In this case, Saltol, badh2 (aroma) and GS3 (grain length) were all selected at the same time by MAS. Lines such as Saltol-introgressed Pusa Basmati have maintained acceptable yield in trials at Ece ≈ 6 dS m^−1^ while retaining Basmati aroma and grain morphology [161]; confirmation across seasons and sites is still required.

### 5.3. Genomic Selection and Trait Discovery

Advancements in genotyping technology have allowed for GWAS and Genomic Selection (GS) to dissect the polygenetic architecture of salt tolerance in a rapidly declining cost. In addition to the major QTLs (such as Saltol), numerous minor-effect loci also control tolerance. GWAS has been conducted on a variety of rice panels to identify SNPs associated with ionic homeostasis, survival and yield under stress. For example, Campbell et al. [159] identified loci on chromosomes 2 and 7 for seedling vigor, revealing novel transporters and transcription factors overlooked in earlier biparental mapping [136,159].

GS predicts breeding values using genome-wide markers, serving as a useful tool when phenotyping is costly or destructive. Moderate accuracy for salt tolerance was demonstrated in an IRRI pilot study that showed early-generation selection and reduced the resource requirement for testing of large populations [162]. Despite this, the challenge of using GS to predict salinity remains because of the interactions between the genotype and environment (G × E), and the strong G × E interaction necessitates data from multiple environments to develop a robust model that incorporates phenotypic plasticity. Also, with the speed-breeding system (off-season nurseries and MAS), tolerance locus fixation can be achieved through the fast cycling of several generations per year.

### 5.4. Gene Editing and Transgenic Approaches

New genetic engineering technologies complement breeding by enabling direct manipulation of specific genes for tolerance or quality. These approaches can be grouped into two broad categories: transgenic overexpression of tolerance-related genes and CRISPR/Cas9-based targeted editing.

#### 5.4.1. Transgenic Overexpression Approaches

Early studies primarily focused on the gene of *AtNHX1* (vacuolar Na^+^/H^+^ antiporter of *Arabidopsis*), which imparted only moderate salt tolerance [163]. Later, researchers tried to constitutively overexpress endogenous homologs such as *OsNHX1* and *OsSOS1*. However, this often caused yield reduction under normal conditions. These fitness costs are minimized by targeting current strategies with the use of stress-inducible promoters (from dehydrins or the *rd29A* gene). This restricts the gene activation to stress time. The superior method has consequently yielded much higher survival and yield in salt-stress conditions, without any loss of normal growth in non-stressed conditions [164].

Researchers have overexpressed several transcription factors (TFs), such as *SNAC1* and *OsDREB1A*, to improve stress tolerance. Particularly, rice overexpressing *SNAC1* showed better performance than wild-type under drought and mild salinity conditions. Importantly, it showed no yield reduction under normal conditions, marking a rare success in balancing stress resilience with productivity [106]. In general, an overexpression of *OsbZIP72* induced salt tolerance in plants via its activation of ABA signaling pathways [165]. Transgenic plants with OSAlaAT also showed beneficial effects on nitrogen metabolism under saline stress, apart from TF regulation. In contained saline-soil trials, this led to approximately 20% higher grain yield [166]; the advantage has not yet been verified in multi-environment field trials.

Researchers have introduced genes for osmolytes that rice does not synthesize naturally, aiming to enhance osmotic adjustment. For instance, the bacterial *codA* gene (glycine betaine synthesis) was transformed into rice. This led to the accumulation of betaine and enhanced salinity tolerance, leading to an increase in biomass of around 20% in saline conditions [75]. Other approaches involved the production of osmoprotectants, including genes for biosynthesis of mannitol (mtlD) and trehalose-6-phosphate synthase (*TPS*). In addition, it was reported that a multi-gene stacking strategy using *codA* and barley hva1 (encoding an LEA protein) and a halophytic *NHX* gene led to a high degree of tolerance [80]. However, the complexity of stacking various transgenes remains a major challenge.

#### 5.4.2. CRISPR/Cas9 Gene Editing

CRISPR has been used to delete negative regulators of stress. A typical example is the *GS3/AT1* gene. The best-characterized alkaline-tolerance locus, AT1, was identified in sorghum, where loss of function enhances growth under alkaline pH through G-protein signaling and altered aquaporin activity [48]. The rice homologue is GS3, a well-established grain-length gene. Rice gs3 loss-of-function mutants show longer grains, and there are reports that they also perform better under sodic conditions. However, this inference is largely indirect, being transferred from the sorghum AT1 phenotype, and the alkaline-tolerance effect of GS3 in rice has not yet been demonstrated consistently across genetic backgrounds or in multi-environment field trials. We therefore describe GS3 as a plausible dual-function target whose alkalinity benefit in rice remains to be confirmed. MutMap was another example that discovered an OsRR22, a negative regulator in cytokinin signaling. Loss of function mutations in OsRR22 have been found to enhance the salt-stress tolerance of root growth. CRISPR editing of this gene in elite varieties is underway to copy the tolerance seen in the Japanese cultivar Hitomebore Salt Tolerant [167]. In addition to gene knockout, promoter editing is another method for controlling in situ key transporters (*HKT1*;5, *SOS1*) without pleiotropic trade-offs.

For grain quality, gene editing fixes defects in stress-tolerant lines. For instance, high-amylose genotypes with low eating quality were improved by making *Wx* allelic variants via CRISPR to adjust starch composition [168]. Conversely, editing *BADH2* to generate aroma could raise the market value of tolerant lines. Because badh2 loss of function abolishes glycine betaine synthesis, a potential trade-off between aroma and osmoprotection cannot be excluded, although direct field evidence for such a trade-off is currently lacking. This possibility should be tested explicitly rather than assumed. Theoretically, multiplex CRISPR editing can be used to introduce more than one desirable allele, increase the activity of positive regulators, knock out suppressors, and improve quality gene promoters in a single background. This is an important new area for crop development that relies on customization.

### 5.5. Translational Challenges: From Controlled Conditions to Field Breeding

Although controlled-environment studies have greatly advanced our mechanistic understanding, their direct translation to breeding programs and field environments faces several major obstacles. First, most laboratory and greenhouse experiments impose a uniform, acute stress that rarely mirrors the spatially and temporally heterogeneous mosaics of salinity, alkalinity, and sodicity found in farmers’ fields. Second, genotype rankings obtained in hydroponic or pot trials often differ from those observed under natural field conditions, because soil–root interactions, microbial communities, and water management modify stress expression in ways that cannot be reproduced in simplified systems. Third, many candidate genes and QTLs have been validated only against specific genetic backgrounds; their effects can be masked by epistasis, phenology, and genotype-by-environment interactions. Finally, the cost and labor required for multi-environment field testing remain the most significant bottleneck in breeding programs. We therefore recommend that future research combine controlled validation of individual mechanisms with field-based multi-stress screening, and that genomic prediction models incorporate physiological prescreening data to bridge the gap between mechanistic research and practical variety development.

Evidence hierarchy and readiness levels. The breeding strategies reviewed above rest on heterogeneous evidence. We therefore distinguish three tiers: (i) direct field evidence in rice (e.g., Saltol/FL478 introgression lines tested across sites); (ii) indirect or cross-species evidence (e.g., sorghum AT1 as the basis for rice GS3; heterologous BADH overexpression); and (iii) controlled-environment or transgenic evidence (e.g., greenhouse sodic-growth tests of gs3 mutants, hydroponic validation of OsMADS25). Claims of application readiness should be restricted to tier (i). Genotypes supported only by tiers (ii) and (iii) should be described as candidates requiring multi-environment, multi-stress field validation, ideally with concurrent phenotyping for yield, grain quality and stress-type-specific traits.

## 6. Conclusions and Prospective

Saline–alkaline stress severely limits rice yield and quality. The seedling and reproductive stages are most vulnerable. Diagnostic thresholds (soil ECe and pH; Table 1) help classify field conditions. Yield losses are caused primarily by reduce tillering, spikelet sterility, and impaired grain filling. Quality deterioration is manifested by an increased chalkiness and protein/starch ratio under moderate to severe stress (mild stress may improve some traits in some genotypes), and it is related to the interruption of starch-protein deposition. Mechanistically, the stress tolerance pathways rely on different pathways. These include ion homeostasis (mediated by *OsHKT1;5*, *OsSOS1*, *OsNHX1*, and *OsAKT1*), osmotic adjustment, hormone crosstalk, ROS scavenging, and photosynthetic protection. Sustainable mitigation requires combining agronomic reclamation with genetic improvement. Agronomic measures include leaching, gypsum/FGD-gypsum, organic or biochar amendments, and PGPR. Genetic improvement ranges from *Saltol*/MAS pyramiding to genomic selection and CRISPR/Cas9 editing of dual-function loci (*GS3/AT1*, *Wx*, *BADH2*). Together, these approaches offer a plausible route to combined yield and quality resilience on marginal lands, although field-validated deployment remains limited and requires multi-environment testing.

A key priority is to move beyond treating salinity and alkalinity as a single stress. Future work should separately resolve salinity-specific (Na^+^/Cl^−^ toxicity, osmotic stress, SOS/HKT/NHX) and alkalinity-specific (rhizosphere acidification, Fe/Zn deficiency), and shared (osmotic-adjustment, antioxidant-defense, hormone-signaling) mechanisms. A central message of this review is that salinity, alkalinity, and sodicity are physiologically and agronomically distinct stresses and should not be pooled into a single ‘salt stress’ category. Each stress imposes different primary constraints, activates different adaptive mechanisms, and requires different management and breeding approaches. The commonly used umbrella term ‘saline–alkali stress’ should therefore be used with caution. We strongly recommend that future research adopts a tripartite framework in which the three stresses are evaluated both independently and in combination, and we hope that the comparative organization of this review provides a useful template for such integrated work.

Future research should be directed towards the following: (1) cloning of stage-specific and alkalinity-specific genes (e.g., *Saltol2, OsMADS25-AT1 axis*) for precise MAS; (2) multiplex CRISPR editing with promoter tuning for stacking tolerance and quality alleles without pleiotropic penalties; (3) addressing trait trade-offs (e.g., *BADH2* aroma vs. betaine based tolerance; field-level yield cost of constitutive antiporter); (4) improving genomic selection using multi-environment data and speed breeding to overcome G × E limitations; (5) mining of wild relatives (*Porteresia*) and multi-omics for novel alleles; and (6) scaling up the synergies of microbiome amendment to precise decision support models for field-level reclamation.

## Data Availability

The data presented in this study are available on request from the corresponding author.
